# EGFR-targeted gadolinium contrast agents for enhanced molecular magnetic resonance imaging of tumors

**DOI:** 10.1016/j.jbc.2025.110992

**Published:** 2025-11-29

**Authors:** Mengyao Chen, Jing Chen, Xue Ren, Chunping Liu, Chenwu Bai, Xiaoya Wang, Jianli Duan, Shibin Ai, Xinxin Yan, Fan Yang, Xin Liu

**Affiliations:** 1 Department of Colorectal and Anal Surgery, Zhongnan Hospital of Wuhan University, School of Pharmaceutical Sciences, Wuhan University, Wuhan, China; 2 Key Laboratory of Combinatorial Biosynthesis and Drug Discovery, Ministry of Education, Wuhan University, Wuhan, China; 3 Department of Radiology, Union Hospital, Tongji Medical College, Huazhong University of Science and Technology, Wuhan, China; 4 Hubei Provincial Clinical Research Center for Precision Radiology & Interventional Medicine, Wuhan, China; 5 Hubei Key Laboratory of Molecular Imaging, Wuhan, China

**Keywords:** epidermal growth factor receptor, peptide, Gd chelate, targeting contrast agent

## Abstract

Magnetic resonance imaging (MRI) is a widely used tool for tumor diagnosis, but current contrast agents often lack sufficient sensitivity and specificity. To address these limitations, we developed novel gadolinium-based (Gd) contrast agents conjugated with epidermal growth factor receptor (EGFR)-binding peptides (EBPs) for targeted MRI of EGFR-overexpressing tumors. The synthesized EBP-Gd-DO3A and EBP-(Gd-DO3A)_3_ conjugates exhibited high binding affinity and specificity for EGFR-positive cells, as demonstrated through cell binding assays and fluorescence microscopy. Competitive binding assays confirmed EGFR-mediated specificity. *In vitro* MRI revealed strong contrast enhancement in EGFR-overexpressing tumor cells, while *in vivo* studies in xenograft models showed increased tumor uptake and prolonged contrast retention. These agents demonstrated superior specificity and sensitivity compared to traditional non-targeted Gd-DO3A, offering a promising approach for improved tumor detection, grading, and prognosis in clinical molecular imaging.

Magnetic resonance imaging (MRI) is a powerful technique that plays an important role in the diagnosis, staging, and follow-up of tumors (1, 2, 3). Since low-molecular-weight gadolinium chelates (Gd-chelates), such as Dotarem (Gd-DOTA), ProHance (Gd-HP-DO3A), and Gadovist (Gd-DO3A-button), can enhance image contrast in the surrounding tissues (4), they have been approved for medical use in many MRI applications (1, 5). However, these Gd chelates are usually nonspecific extracellular contrast agents that are characterized by high vessel permeability, rapid excretion, and transient blood and tissue retention (6), leading to poor specificity in distinguishing malignancies and benign tumors with hypervascularity and unsatisfactory sensitivity for some early-stage cancers (7, 8).

To overcome the low sensitivity of MRI for tumors, a number of targeted MRI contrast agents have been studied. For example, various delivery systems, such as antibodies, liposomes, colloids, nanoparticles, and polymer conjugates with a high payload of contrast agents, have been prepared to increase the local concentration of the agents at the target sites. However, these macromolecular contrast agents do not display significantly improved tumor targeting due to their large size and related biophysical characteristics (extended residence in the bloodstream, slow tumor penetration, and slow blood clearance) (9). Given their slow and incomplete elimination, the release and the accumulation of free Gd ions in the body may cause toxic side effects, such as nephrogenic systemic fibrosis (10).

Compared with macromolecules, peptide-targeted contrast agents are smaller and readily diffuse into tumor tissues, enabling effective cancer molecular imaging. The small size also allows unbound contrast agents to be excreted from the body *via* the renal route to reduce the background signal and minimize the accumulation of Gd chelates in the body. Recently, a number of target-specific MRI contrast agents designed to bind to overexpressed receptors of tumor cells could drastically improve this imaging process due to the *in situ* accumulation of the MRI contrast agents. For example, Gd-DOTA was conjugated to small peptides to target extra domain B fibronectin, which resulted in robust tumor contrast enhancement in MRI of the PC3 mouse prostate cancer model (10).

Overexpression of epidermal growth factor receptor(EGFR) has been consistently observed in many human epithelial cancers, including gastric, colorectal, bladder, pancreatic, ovarian, lung, and breast cancers (11), making EGFR a validated target for cancer diagnosis and therapy (12, 13). In tumor cells, the overexpression of EGFR has been shown to be closely related to the malignancy, metastasis, and angiogenesis (14, 15), thus making EGFR a promising binding target for MRI contrast agents. EGF is a major important ligand for EGFR, and it binds to this receptor with high specificity and affinity (16). The CMYIEALDKYAC sequence within EGF has been identified as a key receptor-binding domain of EGFR and designated as the EGFR-binding peptide (EBP) (17). Because of the features of EBP binding to EGFR and the overexpression of EGFR in a wide variety of tumors, we previously designed a cytotoxic analogue that links doxorubicin (DOX) to EBP *via* an ester bond at DOX position 14 through a glutarate spacer and demonstrated that this conjugate had high affinity and specificity for EGFR-overexpressing tumor cells, resulting in enhanced antitumor efficacy and reduced systemic toxicity (18, 19). Therefore, EBP has become a promising vector for EGFR-targeting Gd chelates to improve the specificity and sensitivity of contrast-enhanced MRI in clinical tumor diagnosis, enabling the application of molecule-targeted drugs *in vivo* for noninvasive tumor subtyping and treatment.

In this study, we synthesized EBP (NH2-CMYIEALDKYAC-COOH) and two EGFR-targeted agents, EBP-Gd-DO3A and EBP-(Gd-DO3A)_3_, bearing one or three Gd-DO3A moieties. The cellular binding and endocytosis in EGFR-overexpressing and control cells were evaluated using inductively coupled plasma-mass spectrometry (ICP-MS) and fluorescence microscopy. Finally, the ability of the agents to differentiate EGFR-overexpressing cells *in vitro* and EGFR-positive *versus* EGFR-negative xenograft tumors *in vivo* was evaluated by MRI.

## Results

### Synthesis and characterization

**Figure 1:**
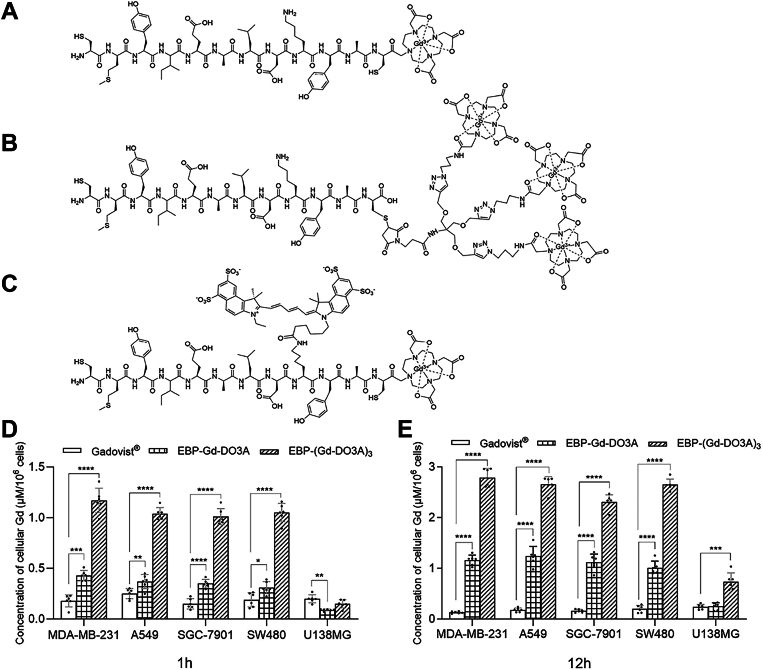
**EBP-Gd-DO3A conjugates: structures and cellular gadolinium.** Schematic structures of (*A*) EBP-Gd-DO3A; (*B*) EBP-(Gd-DO3A)_3_; and (*C*) Cy5.5-EBP-Gd-DO3A. And cells were incubated with EBP-Gd-DO3A, EBP-(Gd-DO3A)_3_, or Gadovist at a Gd concentration of 50 μM for 1 h (*D*) or 12 h (*E*) at 37 °C. The intracellular Gd content was quantified by ICP–MS. Bars represent the mean ± SD. The data represent three independent experiments. Statistical significance was evaluated using one-way ANOVA followed by Tukey’s *post hoc* test. ∗*p* < 0.05, ∗∗*p* < 0.01,∗∗∗*p* < 0.001, ∗∗∗∗*p* < 0.0001.

The EBP-Gd-DO3A, EBP-(Gd-DO3A)_3_, and Cy5.5-EBP-Gd-DO3A conjugates were prepared by stepwise conjugation of the CMYIEALDKYAC peptide with DO3A-tris-tert-butyl ester or Cy5.5-NHS ester, followed by deprotection and complexation with Gd(OAc)_3_ in water. These peptide molecular probes are composed of covalently bound Gd-chelate or Cy5.5 dye and a recognition motif (targeting group). All compounds are >95% pure by high-performance liquid chromatography (HPLC) analysis (Figs. S1 and S2). The resultant structures of the conjugates are shown in Figure 1, *A*–*C*. The synthesized agents were characterized by MALDI-TOF with m/z of 1951.8 (observed), 1951.32 (calculated); 3791.9 (observed), 3791.36 (calculated); and 2845.9 (observed), 2845.39 (calculated) for EBP-Gd-DO3A, EBP-(Gd-DO3A)_3_, and Cy5.5-EBP-Gd-DO3A, respectively (Fig. S3). The Gd content determined by ICP-MS was 7.45% (w/w) in EBP-Gd-DO3A and 11.49% (w/w) in EBP-(Gd-DO3A)_3_. Both the longitudinal (*r*_*1*_) and transverse (*r*_*2*_) relaxivities of the complexes, as well as those of the control Gadovist, measured in water on 3T MRI at 37 °C, were defined and plotted as a function of their Gd concentration, as presented in Figure S4. The *r*_*1*_ and *r*_*2*_ of Gadovist are 3.14 and 3.85 mM^-1^s^-1^, respectively, in agreement with those of commercial Gadovist (20). However, both the *r*_*1*_ and *r*_*2*_ of EBP-Gd-DO3A were 5.08 and 5.44 mM^-1^s^-1^ and 7.24 and 7.62 mM^-1^s^-1^ for EBP-(Gd-DO3A)_3_, respectively. The conjugation of the peptide to Gd-DO3A exhibited higher relaxivities (*r*_*1*_ and *r*_*2*_) than the commercially available Gd-DO3A contrast agent (Gadovist) under the same conditions, as frequently reported in the literature (21).

### Accumulation of both conjugates in EGFR-overexpressing tumor cells

The baseline EGFR expression levels of the five tumor cell lines MDA-MB-231, A549, SGC-7901, SW480, and U138MG were confirmed by Western blot analysis (Fig. S5). The receptor-binding affinity of both conjugates was evaluated using different EGFR-overexpressing cells. The results indicated no difference in the cellular Gd levels in all of the tested cell lines after incubation with Gadovist for 1 h. However, there were significantly lower Gd levels in the non-EGFR-overexpressing cells (U138MG) after incubation with EBP-Gd-DO3A and EBP-(Gd-DO3A)_3_ compared to those in the EGFR-overexpressing cells. Comparable, Gd concentrations were also observed after 12 h of incubation with either Gadovist or the conjugates (Fig. 1, *D* and *E*, Table S1). These data demonstrated distinct cellular binding of the conjugates from that of Gadovist.

### Binding specificity of EBP peptide

**Figure 2:**
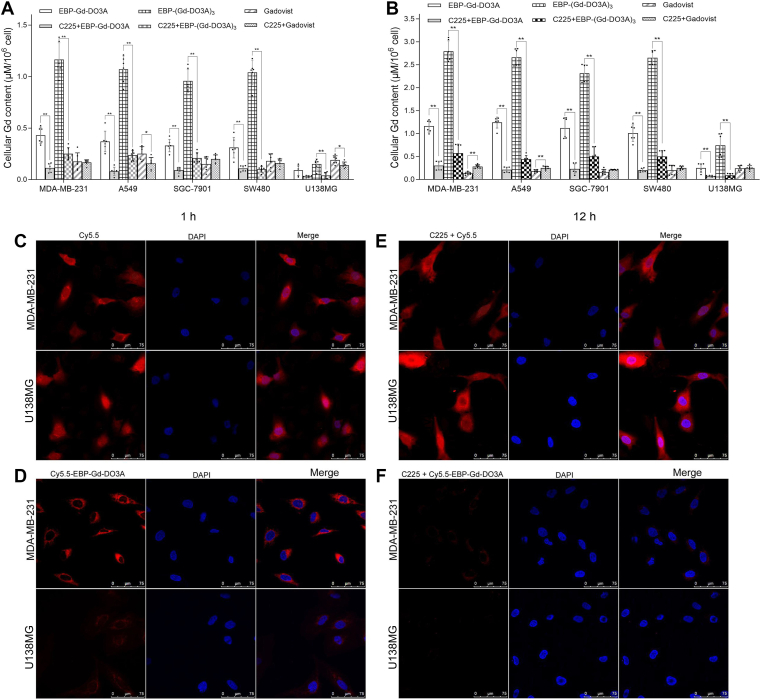
**Cellular Gd accumulation of contrast agents in tumor cells.** MDA-MB-231, A549, SGC-7901, SW480, and U138 MG cells were incubated with EBP-Gd-DO3A, EBP-(Gd-DO3A)_3_ or Gadovist for 1 h (*A*) and 12 h (*B*). The cellular Gd contents were determined by ICP-MS and are presented as 50 nM Gd/10^6^ cells. The data are presented as the mean ± SD of 6 samples for each cell line. For statistical analysis, Mann-Whitney test was used for (*A*) and (*B*). ∗*p* < 0.05; ∗∗*p* < 0.01 (with vs without C225 pretreatment). Confocal fluorescence microscopy images displaying the cellular localization of the Cy5.5 (*C*) and Cy5.5-EBP-Gd-DO3A (*D*) in MDA-MB-231 and U138 MG cells after 1 h of exposure to the agent. The cells were treated with Cy5.5 or Cy5.5-EBP-Gd-DO3A for 1 h and visualized under a fluorescence microscope. Binding and endocytosis of the Cy5.5-labelled EBP-Gd-DO3A conjugate in MDA-MB-231 and U138 MG cells and inhibition of binding by C225. The cells were preincubated with C225 for 12 h and then treated with equimolar concentrations of Cy5.5 (*E*) or Cy5.5-EBP-Gd-DO3A (*F*) for 1 h prior to visualization. Fluorescence microscopy was performed. Merged images display the overlay of nuclear DAPI staining (*blue*) and Cy5.5 dye (*red*).

The cells were preincubated with the anti-EGFR antibody cetuximab (C225) for 12 h and then treated with EBP-Gd-DO3A, EBP-(Gd-DO3A)_3_ or Gadovist. No significant difference in the cellular Gd levels was observed in any of the tested cell lines pretreated with or without C225 after 1 h of treatment with Gadovist. However, after 1 h of incubation with either conjugate, the cellular Gd accumulation was reduced in all the cells that were preincubated with C225 compared to the corresponding cells that were not pretreated with C225 (Fig. 2*A*). A similar trend was observed following 12 h treatments with EBP-Gd-DO3A, EBP-(Gd-DO3A)_3_ or Gadovist after C225 preincubation (Fig. 2*B*), demonstrating the inhibition of the binding of EBP-Gd-DO3A and EBP-(Gd-DO3A)_3_ to cells by C225 and thus the involvement of EGFR in the cellular delivery of the conjugates. The competitive binding assay results demonstrated the EGFR-specific binding affinity and specificity of both conjugates.

To further verify the affinity and specificity of the agents, EGFR-overexpressing MDA-MB-231 cells and non-EGFR-overexpressing U138MG cells were incubated with Cy5.5 or Cy5.5-EBP-Gd-DO3A and evaluated by fluorescence microscopy. As shown in Figure 2, fluorescence from Cy5.5 was observed in the cytoplasmic and perinuclear regions of both the MDA-MB-231 and U138 MG cells after 1 h of incubation. The Cy5.5 fluorescence intensity was also the same in both the MDA-MB-231 and U138MG cells, as assessed by Image-Pro Plus software (Fig. 2*C*). After the cells were incubated with the Cy5.5-EBP-Gd-DO3A conjugate for 1 h, the red fluorescence was clearly present in the cytoplasmic and perinuclear regions of both cell lines, but the fluorescence intensity in the EGFR-overexpressing MDA-MB-231 cells was much stronger than that in the non-EGFR-overexpressing U138MG cells (Fig. 2*D*).

We next sought to determine the effect of the anti-EGFR antibody C225. Although preincubation of the cells with the anti-EGFR monoclonal antibody C225 did not affect the intracellular distribution of Cy5.5 (Fig. 2*E*), the fluorescence originating from the Cy5.5-EBP-Gd-DO3A conjugate was inhibited by this preincubation. Preincubation with C225 did not affect the fluorescence distribution of Cy5.5 in either cell line but showed an inhibitory effect on the fluorescence intensity when the cells were treated with the Cy5.5-EBP-Gd-DO3A conjugate (Fig. 2*F*). Together, these data clearly demonstrate EGFR-mediated uptake of the Cy5.5-EBP-Gd-DO3A conjugate. The mean fluorescence intensity of the Cy5.5 signal was quantified, and the results are shown in the bar graph in Figure S6.

### MR tumor molecular imaging with EBP-Gd-DO3A and EBP-(Gd-DO3A)_3_

**Figure 3:**
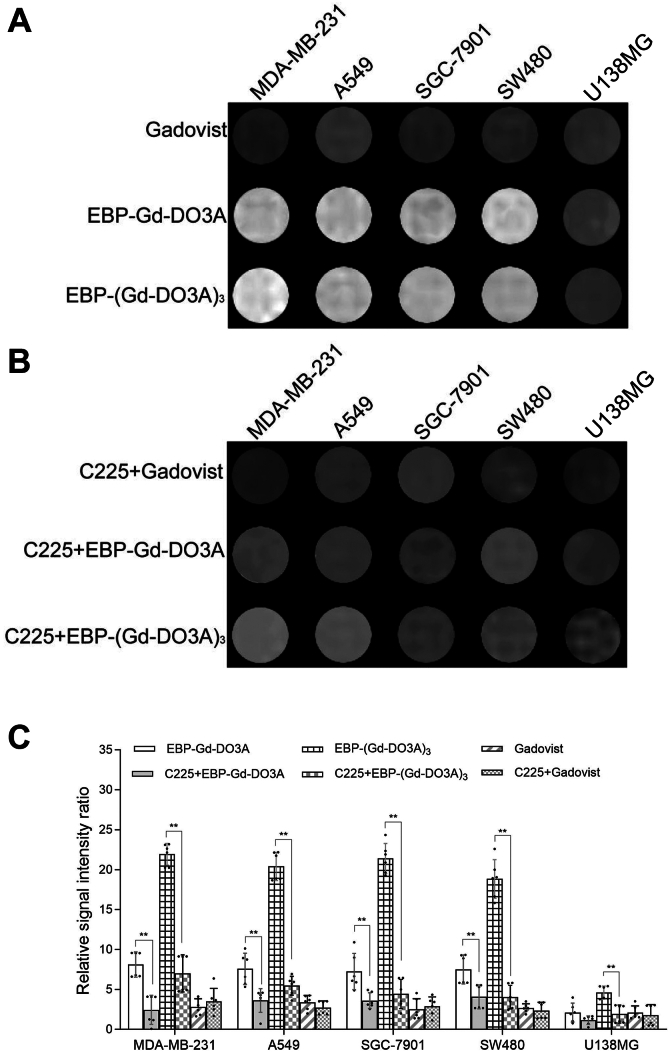
***In vitro*****MR images of tumor cells labelled with contrast agents.** MDA-MB-231, A549, SGC-7901, SW480 and U138 MG cells were incubated with Gadovist, EBP-Gd-DO3A or EBP-(Gd-DO3A)_3_ for 24 h. MRI analysis was conducted by an MR microimaging system. *A*, to detect the effect of the anti-EGFR antibody C225, the cells were preincubated with C225 for 12 h and then labelled with Gadovist, EBP-Gd-DO3A or EBP-(Gd-DO3A)_3_ for 24 h prior to *in vitro* MRI. *B*, Quantitative analysis of relative signal intensity in cells with different EGFR expression levels is shown in (*C*). All data are expressed as the mean ± SD and analyzed by the Mann–Whitney *U* test.∗∗*p* < 0.01, comparison between the indicated groups. The data represent three independent experiments.

To verify whether the targeting contrast agents could significantly increase specificity in MR tumor imaging, *in vitro* contrast-enhanced MRI with EBP-Gd-DO3A and EBP-(Gd-DO3A)_3_ was conducted in different EGFR-expressing cells using Gadovist as a control. The results are shown in Figure 3. The MR enhancement effects were similar for the cells with different EGFR expression levels that were treated with Gadovist (Fig. 3*A*). Quantitative analysis of the ROI showed that the *T*_*1*_ signal intensity was not significantly different in cells with different levels of EGFR expression that were incubated with Gadovist (*p* > 0.05) (Fig. 3*C*). However, after incubation with EBP-Gd-DO3A and EBP-(Gd-DO3A)_3_, strong enhancement effects were observed in the MDA-MB-231, A549, SGC-7901, and SW480 tumor cells, whereas U138MG cell line exhibited much weaker enhancement effect (Fig. 3*A*). A quantitative analysis showed that the signal intensity of the U138 MG cells treated with the targeting agents was lower than that of the other cells, while the difference in the signal intensity between the EGFR-overexpressing cells and non-EGFR-overexpressing cells was statistically significant (∗∗∗*p* < 0.001) (Fig. 3*C*). These results indicated that both contrast agents effectively targeted EGFR-positive tumor cells and could successfully generate significant enhancement effects in MR images.

The EGFR target-specific nature of EBP-Gd-DO3A and EBP-(Gd-DO3A)_3_ was further confirmed by an *in vitro* MR imaging inhibition study. After 24 h of incubation with Gadovist, no significant difference in the enhancement effects in the MR images was observed in all the tested cell lines that were pretreated with or without C225, indicating that EGFR expression was not associated with MR contrast enhancement by Gadovist. On the other hand, after 24 h of incubation with the conjugates, the MRI signal intensity was reduced in all of the cells that were preincubated with C225 (Fig. 3*B*) compared to the MRI signal intensity of the corresponding cells not pretreated with C225 (Fig. 3*A*). Quantitative image analysis revealed that, after incubation with either conjugate, the MRI signal intensity of the C225-preincubated cells (EGFR-overexpressing) showed a significantly greater decrease than that of the cells not preincubated with C225 after incubation with either conjugate (∗∗*p* < 0.01) (Fig. 3*C*).

### Biodistribution in mice

**Figure 4:**
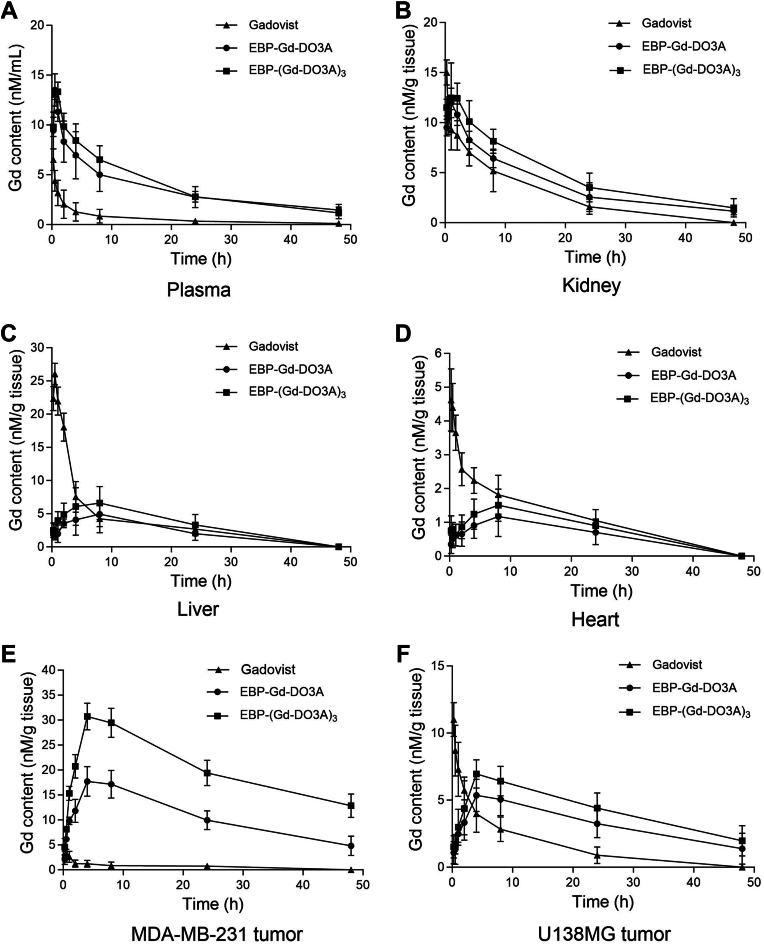
**Biodistribution of contrast agents inMDA-MB-231and U138 MGtumor-bearingmice.** The animals were injected with a single dose of Gadovist (▲), EBP-Gd-DO3A (•) or EBP-(Gd-DO3A)_3_ (▪) at 50 μM Gd/kg, and blood and tissue samples were collected at the indicated times. The Gd concentration was determined in the plasma (*A*), kidney (*B*), liver (*C*), heart (*D*), MDA-MB-231 tumor (*E*), and U138MG tumor tissues (*F*) by ICP-MS after contrast agent administration.

To investigate the *in vivo* biodistribution of the contrast agents, following a single i.v. injection of the contrast agents, blood and tissue samples were harvested from SCID mice bearing subcutaneous MDA-MB-231 (EGFR-overexpressing) and U138 MG (non-EGFR-overexpressing) tumor xenografts at different times, and the Gd levels were measured by ICP-MS. The conjugation of EBP to Gd-DO3A in EBP-Gd-DO3A and EBP-(Gd-DO3A)_3_ led to a significant change in their biodistributions compared to that of the parent molecule Gd-DO3A. As shown in Figure 4*A*, Gadovist rapidly disappeared from circulation, whereas the synthesized conjugates were maintained at a high level for a much longer time. As the primary pathway for the clearance of gadolinium-based contrast agents, the kidney contained the highest Gd level among all the organs tested. For Gadovist, the Gd content in the kidneys decreased from 10.70 ± 2.10 nM/g tissue at 0.5 h to 8.73 ± 1.37 nM/g tissue at 2 h and to 1.58 ± 0.72 nM/g tissue at 24 h (Fig. 4*B*). For EBP-Gd-DO3A, the Gd content in the kidneys was as high as 10.76 ± 1.14 nM/g tissue at 2 h postinjection and decreased to 8.25 ± 0.75 nM/g tissue at 4 h postinjection and to 2.52 ± 0.68 nM/g tissue at 24 h. EBP-(Gd-DO3A)_3_ uptake in the kidneys was 12.40 ± 1.60 nM/g tissue at 2 h, which declined to 8.12 ± 1.28 nM/g tissue at 8 h and to 3.50 ± 1.40 nM/g tissue at 24 h. The substantial uptake of the Gd contrast agents in the kidneys indicated that these compounds were mainly excreted through the renal route. With regard to Gd retention in the liver and heart of the mice, the Gadovist concentration was relatively high at all time points, compared with the EBP-Gd-DO3A and EBP-(Gd-DO3A)_3_ concentrations (Fig. 4, *C* and *D*). The liver and heart uptake values between EBP-Gd-DO3A and EBP-(Gd-DO3A)_3_ were comparable and did not significantly differ.

It is well known that high and persistent tumor uptake of Gd is a concern when designing receptor-targeted contrast agents. After the EBP-Gd-DO3A or EBP-(Gd-DO3A)_3_ conjugate was injected, the Gd content in the MDA-MB-231 tumor tissues increased with time during the first 2 h and peaked at 17.70 ± 2.10 nM/g tissue or 30.70 ± 1.90 nM/g tissue at 4 h and decreased to 9.95 ± 1.85 nM/g or 19.42 ± 1.76 nM/g tissue at 24 h (Fig. 4*E*). A significant difference was noted in the values between the MDA-MB-231 and U138 MG tumors (Fig. 4, *E* and *F*), suggesting greater Gd retention in the EGFR-overexpressing tumors after injection of EBP-Gd-DO3A at all time points. (Fig. 4*E*). On the other hand, Gadovist uptake in the MDA-MB-231 tumors was 3.36 ± 0.91 nM/g tissue at 0.5 h, which declined to 1.26 ± 0.46 nM/g tissue at 2 h, and no detectable Gd was observed at 24 h. Similarly, the Gd content in the U138 MG tumors declined from 8.70 ± 1.24 nM/g tissue at 0.5 h to 5.76 ± 0.45 nM/g tissue at 2 h and to 0.90 ± 0.23 nM/g tissue at 24 h for Gadovist (Fig. 4*F*). These results suggested that the targeted agents displayed higher tumor uptake than the nontargeted agents.

### MR tumor molecular imaging in mice

**Figure 5:**
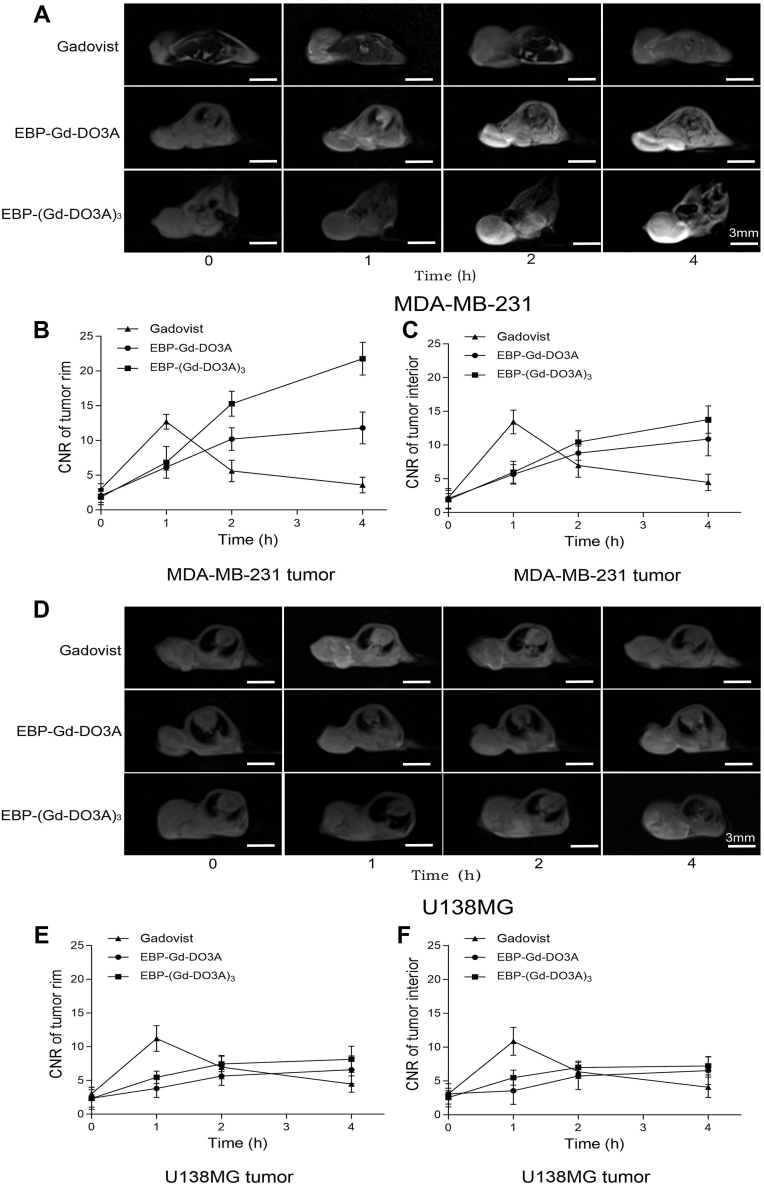
***In vivo* MRI of EGFR positive and negative tumor xenografts.***In vivo* MR images of MDA-MB-231 (*A*) and U138MG (*D*) tumor xenografts in SCID mice (n = 6) before and after intravenous injection of Gadovist, EBP-Gd-DO3A, and EBP-(Gd-DO3A)_3_ at 50 μM Gd/kg. Quantitative analysis of CNR in MDA-MB-231 tumors before and after injection at the tumor edge (*B*) and interior (*C*), and in U138MG tumors before and after injection at the tumor edge (*E*) and interior (*F*).

To test the effectiveness of the synthesized agents as targeted molecular probes in detecting EGFR-positive tumors, *in vivo* tumor-targeting MR imaging of the contrast agents was performed on SCID mice bearing subcutaneous MDA-MB-231 and U138 MG tumor xenografts at different times. The pre-and postinjection MR images are shown in Figure 5. The signal intensity of the Gadovist-injected mice demonstrated a significant increase for up to 1 h after injection and persisted for 2 h, suggesting that Gadovist has a short imaging window. No significant differences between the MDA-MB-231 and U138 MG tumors were observed for Gadovist, as measured by the contrast-to-noise ratio (CNR) (Fig. 5), indicating that Gadovist was not capable of specifically targeting the EGFR-positive tumors. However, for the MDA-MB-231 tumor xenograft mice administered EBP-Gd-DO3A and EBP-(Gd-DO3A)_3_, significant enhancement was observed in the tumor tissues at 2 h postinjection, and strong enhancement remained for at least 4 h after administration (Fig. 5*A*). The targeting agents resulted in more prolonged contrast enhancement, possibly due to EGFR binding. The CNR in the tumor border or interior before and after the injection of contrast agents was measured and plotted in Figure 5 to quantitatively analyze the MR signal intensity. The targeting agent yielded a CNR that was approximately twice as high as that of Gadovist in the tumor border at the same dose (Fig. 5, *B* and *C*). Compared to those in the MDA-MB-231 tumors, weaker contrast enhancement and lower CNR were observed in the U138 MG tumors injected with EBP-Gd-DO3A and EBP-(Gd-DO3A)_3_ (Fig. 5, *D*, *E* and *F*). By comparison, EBP-(Gd-DO3A)_3_ resulted in stronger contrast enhancement than EBP-Gd-DO3A, especially in the MDA-MB-231 tumors.

## Discussion

MRI is advantageous due to its high spatial resolution of soft tissues and the absence of ionizing radiation (22). The key to effective clinical MR cancer molecular imaging is the design and development of safe, effective targeted MRI contrast agents. This study has shown that it is possible to achieve effective MR cancer molecular imaging by utilizing a small molecule targeted contrast agent that binds properly to selected molecular targets. Overexpression of EGFR has been observed in a variety of cancers, allowing the binding of sufficient small molecule Gd-based contrast agents to generate robust contrast enhancement and to overcome the low sensitivity of MRI (23, 24).

To efficiently target EGFR-overexpressing tumor cells, the conjugation of Gd-DO3A to EBP must preserve the binding ability to EGFR. Cell binding assays demonstrated the binding affinity of the synthesized agents in EGFR-overexpressing cells. As shown in Figure 1, *D* and *E*, the cellular Gd level in both EGFR-overexpressing and non-EGFR-overexpressing cells was almost the same after the addition of Gadovist, as determined by ICP-MS; in contrast, the cellular Gd level in EGFR-overexpressing cells was much higher than that in non-EGFR-overexpressing cells after incubation with EBP-Gd-DO3A and EBP-(Gd-DO3A)_3_. Fluorescence microscopy further demonstrated the high affinity of the conjugates for EGFR-overexpressing cells: the fluorescence intensity in the MDA-MB-231 cells was much higher than that in the U138 MG cells after incubation with the Cy5.5-EBP-Gd-DO3A conjugate (Fig. 2). Additionally, observations of EGFR-overexpressing and non-EGFR-overexpressing cells by MR imaging experiments also revealed high targeted specificity for EGFR of both the EBP-containing contrast agents (Fig. 3). These data indicate that the binding ability of EBP to EGFR was preserved in the synthesized conjugates.

Based on *in vitro* competitive binding assays, the results indicate that the synthesized agents have high targeted specificity toward EGFR-overexpressing cells. As shown in Figure 2, the competitive anti-EGFR monoclonal antibody C225 could significantly reduce the binding of EBP-Gd-DO3A and EBP-(Gd-DO3A)_3_ to EGFR, leading to decreased cellular Gd levels of both conjugates in tumor cells. Fluorescence microscopy experiments performed with EGFR-overexpressing (MDA-MB-231) and non-EGFR-overexpressing (U138 MG) cells also showed that pretreatment of the cells with C225 inhibited Cy5.5-EBP-Gd-DO3A binding and subsequent internalization (Fig. 2), which is indicative of EGFR-mediated endocytosis, confirming the specificity of the targeted agent for EGFR. MR imaging further showed that the contrast-enhancement effect of the synthesized agents in both EGFR-overexpressing and non-EGFR-overexpressing cells became very weak after preincubation with C225 (Fig. 3*B*), demonstrating the inhibition of conjugate binding to cells. These results suggest that the specific effect of the conjugates was mediated through EGFR. In addition, it has been demonstrated that the uptake of Gadovist by cells is nonspecific. These results are in agreement with our original hypothesis that the combination of EBP can create a more efficient targeting contrast agent and can improve tumor detection rates.

Notably, the improved sensitivity of small-molecule-targeted contrast agents is key for effective MR cancer molecular imaging (25, 26). First, the high sensitivity of the targeted contrast agents is attributed to the significant increase in the Gd content in the tumors, as demonstrated by the results in Figure 4. A significant increase in the Gd content in targeted tumors is expected to enhance both the sensitivity and specificity of clinical tumor imaging. Second, as shown in Figure 4, there was a significantly higher Gd level in the MDA-MB-231 (EGFR-overexpressing) tumors and lower Gd levels in all other organs compared to the kidney, which routinely shows higher uptake due to renal clearance. Compared with the traditional Gadovist, both EBP-Gd-DO3A and EBP-(Gd-DO3A)_3_ showed lower Gd accumulation concentration in liver and heart, suggesting that the synthesized targeted contrast agents are more specific to the tumor site, and have less impact on non-target organs (Fig. 4). Consistent with small hydrophilic peptide–Gd agents, the kidney exhibited the highest Gd levels over time for all probes, with lower liver and heart uptake for EBP-Gd conjugates than for Gadovist (Fig. 4, *B*–*D*). These data are consistent with renal elimination being the predominant clearance route. Moreover, as expected in Figure 5, the targeted agents resulted in greater and longer enhancement in the tumor tissues compared to that caused by a nontargeted commercial agent. Taken together, these data demonstrate that the synthesized contrast agents exhibited improved tumor specificity and enhanced imaging performance. Although safety was not directly evaluated in this study, future work will include cytotoxicity assays and *in vivo* safety evaluation to fully characterize the biocompatibility of these agents prior to potential clinical application.

**Figure 6:**
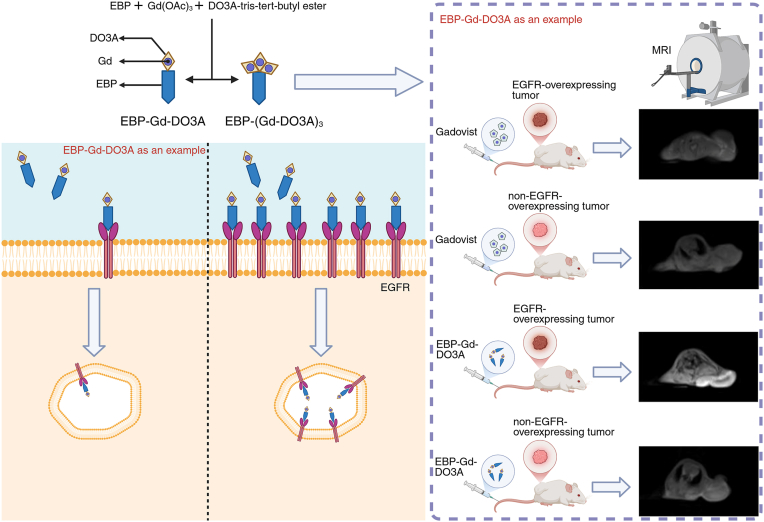
**Schematic representation ofEBP-Gdconjugates and MRI imaging in tumor models.** The figure illustrates the synthesis and application of EBP-Gd-DO3A and EBP-(Gd-DO3A)_3_ conjugates for targeted imaging of EGFR-overexpressing tumors. On the left, the chemical synthesis pathway of EBP-Gd-DO3A and EBP-(Gd-DO3A)_3_ is shown, with EBP conjugated to Gd. The middle section depicts the targeting mechanism, where the EBP conjugates specifically bind to the EGFR on the cell surface, facilitating internalization. On the right, *in vivo* imaging results are shown, where mice bearing EGFR-overexpressing tumors were injected with either Gadovist or EBP-Gd conjugates. MRI images of the tumors are shown, demonstrating enhanced contrast in the EGFR-overexpressing tumors with EBP-Gd-DO3A compared to the non-targeted Gadovist.

In summary, this study has validated the feasibility of EGFR-targeted small molecular contrast agent containing highly stable Gd chelates for effective MR molecular imaging of biomarkers in tumors. As shown in Figure 6, the peptide-targeted contrast agent has shown great potential for accurate tumor and early detection of tumors with contrast-enhanced MRI. MR cancer molecular imaging with the targeted agent also has the potential for cancer grading and prognosis.

## Experimental procedures

### Materials

N, N-Dimethylformamide (DMF), N, N-diisopropylethylamine (DIPEA), and all of the Fmoc-protected amino acids were purchased from GL Biochem (Shanghai, China). 1,4,7,10-Tetraazacyclododecane (cyclen) was purchased from Strem Chemicals (Newburyport, MA). Cy5.5 N-Hydroxysuccinimide ester (Cy5.5-NHS) was purchased from Lumiprobe (Hallandale Beach, FL). The human breast cancer cell line MDA-MB-231, human lung carcinoma cell line A549, human gastric cancer cell line SGC-7901, human colon cancer cell line SW480, and human glioma cell line U138 MG were obtained from the China Center for Type Culture Collection (CCTCC, Wuhan, China). The cells were cultured at 37 °C in RPMI-1640 medium (Gibco BRL, Grand Island, NY) containing 10% fetal bovine serum (HyClone, Logan, UT), 10^5^ U/L penicillin and 100 mg/L streptomycin.

### Synthesis of EBP-Gd-DO3A

DO3A-tris-tert-butyl ester was synthesized from cyclen as described in the literature (27). Azido-(Gd-DO3A) was prepared using modified methods from a previous study (1), and Maleimido-tris-propargyl was synthesized according to Zhou *et al*. (28). Fmoc-protected EBP (Fmoc-NH-CMYIEALDKYAC-COOH) was synthesized *via* solid-phase peptide synthesis using Rink amide methyl-benz-hydryl-amine resin and purified by reversed-phase high-performance liquid chromatography (RP-HPLC, Thermo Fisher Scientific, Ultimate 3000) (29).

For the EBP-Gd-DO3A conjugate, CMYIEALDKYAC was brominated using carbon tetrabromide (CBr_4_) and triphenylphosphine to give the corresponding bromo-intermediate CMYIEALDKYAC-Br. A mixture of CMYIEALDKYAC-Br, DO3A-tris-tert-butyl ester, K_2_CO_3_, and NBu_4_OH was heated at 60 °C for 48 h under N_2_. After cooling, K_2_CO_3_ were removed by filtration, and the solvent was removed by rotary evaporation. Following the removal of the protective functional groups, Gd(OAc)_3_ was added in excess, and 0.01 M NaOH was used to adjust the pH of the solution to pH 5.5 to 6.0. The solution was stirred at 50 °C for 48 h, and EBP-Gd-DO3A was obtained after chelating the free Gd with ethylenediaminetetraacetic acid (EDTA). The purification of the final compound was performed by RP-HPLC.

### Synthesis of EBP-(Gd-DO3A)_3_

The EBP-(Gd-DO3A)_3_ conjugate was synthesized as reported previously (28). EBP and DIPEA were added to a solution of maleimido-tris-propargyl in DMF. The mixture was stirred under N_2_ for 1 h and then poured into cold ethyl ether to precipitate the product. The product was collected, washed with ethyl ether and dried in a vacuum. EBP-tris-propargyl and azido-(Gd-DO3A) were dissolved in a mixed solvent of *t*-BuOH and water. The solution was degassed and stirred under N_2_ at room temperature for 24 h. After the removal of the protective functional groups, excess Gd(OAc)_3_ was used to ensure complete chelation. After completion, the reaction mixture was dialyzed against 0.5% EDTA for 12 h to remove free Gd(III), and then against water for 24 h. The purified product was obtained by RP-HPLC and subsequently lyophilized to yield the final compound.

### Synthesis of Cy5.5-EBP-Gd-DO3A

The chemical conjugation of Cy5.5 to EBP-Gd-DO3A was performed as described previously (30). A solution of Cy5.5-NHS ester in DMF containing 2% DIPEA was added to a stirred solution of EBP-DO3A. The reaction mixture was stirred for 0.5 h at room temperature to yield Cy5.5-EBP-DO3A. For Gd chelation, Cy5.5-EBP-DO3A was metalated with Gd(OAc)_3_ in water at pH 5.5 to 6.0. Cy5.5-EBP-Gd-DO3A was obtained in a manner similar to the synthesis and purification of the EBP-Gd-DO3A conjugate.

### Characterization of purified compounds

The EBP-Gd-DO3A and EBP-(Gd-DO3A)_3_ conjugates were confirmed by matrix-assisted laser desorption/ionization time-of-flight (MALDI-TOF) mass spectrometry (Applied Biosystems) in a linear mode with 2,5-dihydroxybenzoic acid (2,5-DHB) as the matrix. The Gd content in the conjugates was determined by ICP-MS (Thermo Fisher Scientific) (31). The longitudinal (*r*_*1*_) and transverse (*r*_*2*_) relaxivities of the synthesized agents were measured at room temperature and compared with those of the clinical agent Gadovist (32). Serial dilutions of EBP-Gd-DO3A, EBP-(Gd-DO3A)_3,_ and Gadovist were prepared in water at Gd concentrations of 0.5, 0.25, 0.125, 0.0625, 0.03125, and 0 mM. The relaxivity measurements were performed on a 3T MRI scanner (Trio, Siemens). *T*_*1*_ was measured using an inversion recovery method with a variable inversion time ranging from 50 to 1750 ms. *T*_*2*_ was measured using a Carr-Purcell-Meiboom-Gill sequence with 500 echoes collected. The relaxivities (*r*_*1*_ and *r*_*2*_) of the agents were calculated from the slopes of the plots of 1/*T*_*1*_ and 1/*T*_*2*_ *versus* the Gd concentrations.

### Western blot analysis

The expression of EGFR in the tumor cell lines was analyzed by Western blotting (33, 34). The proteins from the cells were isolated in lysis buffer containing 1% phenylmethylsulfonyl fluoride (PMSF, Beyotime Biotechnology). The protein concentrations were determined using a BCA Protein Assay Kit (Beyotime Biotechnology) and equal amounts of total protein (30 μg) were separated by using 8% SDS-PAGE (Beyotime Biotechnology) and transferred onto the polyvinylidene difluoride (PVDF) membrane (Millipore). The PVDF membranes were blocked with 5% (w/v) skim milk in Tris-buffered saline containing 0.1% Tween-20 (TBST) for 2 h at room temperature, and the PVDF membranes were incubated with primary antibodies EGFR (1:1000 dilution, #sc-71033, Santa Cruz Biotechnology, Inc., Santa Cruz, CA), and β-actin (1:4000 dilution, #A5316, Sigma-Aldrich) at 4 °C overnight. Next, the PVDF membranes were washed three times with TBST (10 min each) and incubated with horseradish peroxidase–conjugated secondary antibody(goat anti-mouse IgG-HRP, 1:2000 dilution, #A0216, Beyotime Biotechnology) for 1 h at room temperature. Specific protein bands were visualized using enhanced chemiluminescence (ECL) detection kit (Amersham Biosciences Inc.).

### Binding specificity of the peptides

The binding of EBP-Gd-DO3A and EBP-(Gd-DO3A)_3_ to the receptor on MDA-MB-231, A549, SGC-7901, SW480, and U138 MG cells was compared with that of Gadovist. The cells were seeded into 100 mm × 20 mm cell culture dishes at a density of approximately 1 × 10^6^ cells per dish and incubated with EBP-Gd-DO3A, EBP-(Gd-DO3A)_3_ or Gadovist at a Gd concentration of 50 μM for 1 h or 12 h at 37 °C. After incubation, the cells were washed once with PBS followed by mild acidic buffer (50 mM glycine, 150 mM NaCl (pH 3.0)) at 4 °C for 5 min. Then, the cells were washed twice with PBS, and the cellular Gd content was measured by ICP-MS.

### EGFR inhibition assay

To further verify the receptor-binding affinity and specificity of EBP-Gd-DO3A and EBP-(Gd-DO3A)_3_, a competitive cell-binding assay was carried out. MDA-MB-231, A549, SGC-7901, SW480, and U138 MG cells were preincubated with fresh serum-free medium containing 5 μg/ml anti-EGFR monoclonal antibody C225 (ImClone Systems, Inc., New York, NY) for 12 h at 37 °C. The culture supernatant was removed from the resulting solution, followed by the addition of EBP-Gd-DO3A, EBP-(Gd-DO3A)_3,_ or Gadovist at a Gd concentration of 50 μM for the indicated time points. The cellular Gd content was measured by ICP-MS as described above, and the C225-induced reduction in the cellular Gd accumulation was used to calculate an inhibition index of cellular Gd uptake (34).

### Laser scanning confocal fluorescence microscopy

To investigate the intracellular distribution of EBP-Gd-DO3A in cells with different levels of EGFR expression, MDA-MB-231 and U138 MG cells were seeded on glass coverslips in 24-well plates (5 × 10^4^ cells per well). After 24 h, the cells were washed twice with serum-free medium, treated with 0.3 μM Cy5.5 or Cy5.5-EBP-Gd-DO3A (concentration of equivalent Cy5.5), and incubated at 37 °C for 1 h. Following incubation, cells were washed three times with PBS (pH 7.4) to remove unbound probes and were stained with 4′,6-diamidino-2-phenylindole (DAPI) (Sigma-Aldrich) for 10 min and examined with a laser scanning confocal fluorescence microscope (Leica, TCS SP8) with a 40 × objective lens. The fluorescence signals from Cy5.5 and its conjugates were visualized at λ_ex_ = 680 nm and λ_em_ = 710 nm, while the fluorescence signals of DAPI (nucleus) were performed at λ_ex_ = 350 nm and λ_em_ = 460 nm. The laser power, illumination intensity, and exposure time were kept identical across all samples to ensure quantitative comparability. To further characterize the selective cellular uptake of the conjugate *via* the EGFR-mediated pathway, the cells were preincubated with C225 for 12 h at 37 °C before probe treatment. Following the removal of the pretreatment solution, the cells were washed with serum-free medium, treated with Cy5.5 or Cy5.5-EBP-Gd-DO3A conjugate, and incubated at 37 °C as described above. The cells were stained with DAPI and visualized using the laser scanning confocal fluorescence microscope, and the fluorescent intensity of the cells was analyzed using Image J.

### MR imaging assay

For the *in vitro* MRI assay, MDA-MB-231, A549, SGC-7901, SW480, and U138 MG cells were seeded into 100 mm × 20 mm cell culture dishes at a density of approximately 1 × 10^6^ cells per dish and incubated with EBP-Gd-DO3A, EBP-(Gd-DO3A)_3_ or Gadovist at a Gd concentration of 50 μM for 24 h at 37 °C. After the completion of the labelling, the cells were washed with PBS 3 times and collected into 1.5 mm-i.d. capillary tubes that were centrifuged at 1400 rpm for 5 min to enable cells to be well packed in the bottom of the capillary for the MRI. The *in vitro* MRI experiments were performed on an 11.7 T Bruker NMR spectrometer that functioned as an MR microimaging system with a maximum gradient strength of 1.0 T/m. A linear 15-mm-i.d birdcage coil was used for radiofrequency transmission and signal reception. The parameters used for acquiring images were TR = 500 ms, TE = 5.2 ms, FOV = 12 × 12 mm^2^, matrix = 96 × 96, flip angle = 90°, slice thickness/gap = 0.8/0.2 mm and number of averages = 4.

### *In vitro* MR imaging inhibition assay

The C225-preincubated cells described above were imaged with MRI to observe the contrast enhancement effects. The method used for the MR imaging of cell pellets was the same as that for the *in vitro* MR imaging of the conjugates described above.

### Tumor xenograft model

The 6- to 8-week-old male non-obese diabetic (NOD)/severe-combined immunodeficient (SCID) mice were used to establish MDA-MB-231 (EGFR-overexpressing) and U138 MG (non-EGFR-overexpressing) xenograft models (35). The mice were obtained from the Experimental Animal Center of Tongji Medical College, Huazhong University of Science and Technology, and all the experimental procedures involving animals were further approved by the Animal Care and Use Committee of Huazhong University of Science and Technology. The mice were housed separately in polycarbonate cages and provided with food and water. Tumors were grown under the skin by s.c. injection of 1 × 10^6^-3 × 10^6^ cells. The mice were randomized into groups for the biodistribution assay and MR imaging *in vivo* when the xenografts reached 5 to 7 mm in diameter. All procedures were approved by the Institutional Animal Care and Use Committee of Huazhong University of Science and Technology (approval no.270193).

### *In vivo* biodistribution study

The tumor-bearing SCID mice were randomly divided into groups, with 6 animals in each group, an injected *via* the tail vein with 50 μM Gd/kg EBP-Gd-DO3A, EBP-(Gd-DO3A)_3_, or Gadovist. At the selected time points (0.25, 0.5, 1, 2, 4, 8, 24, and 48 h) after injection, blood was collected and centrifuged to separate the plasma. Then, organ and tissue samples, including the kidneys, liver, heart, and tumor, were collected and weighed. The tissue samples were liquefied for 1 week and then transferred into centrifuge tubes and centrifuged at 14,000 rpm for 5 min. The resulting supernatants were diluted tenfold with deionized water and centrifuged again under the same conditions. The Gd concentration in the samples was measured by ICP-MS (36).

### *In vivo* MR imaging assay

Tumor-bearing SCID mice were randomly grouped and scanned with a Bruker Biospec 7 T MRI scanner (Bruker Corp.) with a volume radio frequency (RF) coil. The mice were anaesthetized with 2% isoflurane with supplemented O_2_ continuously during the whole imaging process *via* a nose cone. A group of 6 mice was used for each agent. After preinjection baseline scan, EBP-Gd-DO3A, EBP-(Gd-DO3A)_3,_ or Gadovist was administered *via* the tail vein at a dose of 50 μM Gd/kg, followed by a saline flush. *T*_*1*_-weighted 2D axial images were then acquired at different time points after the injection for up to 4 h. The parameters of the 2D *T*_*1*_-weighted gradient echo sequence were TR/TE = 151.2/1.9 ms, FOV = 3.0 cm, slice thickness = 1.2 mm, slice number = 20, average = 1, flip angle = 80°, and matrix = 128 × 128 (28, 37).The CNR were calculated with standard methods (CNR = [S_tumor_-S_background_]/SD_noise_).

### Statistical analysis

Statistical analysis was performed using GraphPad Prism version 10.1.2. Data are presented as the mean ± SD. The normality of the data distribution was tested using the Shapiro-Wilk test, and the homogeneity of variances was assessed using the Brown–Forsythe test. For comparisons between two groups, data that did not meet the assumptions of normality or equal variance were analyzed using the Mann–Whitney test, while data satisfying these assumptions were analyzed using a two-tailed Student’s *t* test. For comparisons among three groups, a one-way ANOVA followed by Tukey’s *post hoc* test was applied for data meeting homogeneity of variance. A *p*-value of less than 0.05 was considered statistically significant (∗*p* < 0.05, ∗∗*p* < 0.01, ∗∗∗*p* < 0.001,∗∗∗∗*p* < 0.0001).

## Data availability

The authors confirm that the data supporting the findings of this study are available within this article and as its supplementary materials.

## Supporting information

This article contains supporting information.

## Conflict of interests

The authors declare that they do not have any conflicts of interest with the content of this article.

## Supplementary Table 1 Final

Supplementary Table 1 Final

## References

[1] Mastarone D.J., Harrison V.S., Eckermann A.L., Parigi G., Luchinat C., Meade T.J. (2011). A modular system for the synthesis of multiplexed magnetic resonance probes. J. Am. Chem. Soc..

[2] Leach M.O., Brindle K.M., Evelhoch J.L., Griffiths J.R., Horsman M.R., Jackson A. (2005). The assessment of antiangiogenic and antivascular therapies in early-stage clinical trials using magnetic resonance imaging: issues and recommendations. Br. J. Cancer.

[3] El Beltagi A.H., Elsotouhy A.H., Own A.M., Abdelfattah W., Nair K., Vattoth S. (2019). Functional magnetic resonance imaging of head and neck cancer: performance and potential. Neuroradiol J..

[4] Caravan P. (2006). Strategies for increasing the sensitivity of gadolinium based MRI contrast agents. Chem. Soc. Rev..

[5] Bryant L.H., Jordan E.K., Bulte J.W., Herynek V., Frank J.A. (2002). Pharmacokinetics of a high-generation dendrimer-Gd-DOTA. Acad. Radiol..

[6] Shuvaev S., Akam E., Caravan P. (2021). Molecular MR contrast agents. Invest Radiol..

[7] Cai Z., Jiang L., Cao Y., Fu S., Wang S., Jiang Y. (2024). Lipophilic group-modified manganese(Ii)-based contrast agents for vascular and hepatobiliary magnetic resonance imaging. J. Med. Chem..

[8] Fu S., Cai Z., Liu L., Fu X., Xia C., Lui S. (2023). PEGylated amphiphilic Gd-DOTA backboned-bound branched polymers as magnetic resonance imaging contrast agents. Biomacromolecules.

[9] Banerjee S.R., Ngen E.J., Rotz M.W., Kakkad S., Lisok A., Pracitto R. (2015). Synthesis and evaluation of Gd(III) -Based magnetic resonance contrast agents for molecular imaging of prostate-specific membrane antigen. Angew. Chem. Int. Ed. Engl..

[10] Li Y., Han Z., Roelle S., DeSanto A., Sabatelle R., Schur R. (2017). Synthesis and assessment of peptide Gd-DOTA conjugates targeting extradomain B fibronectin for magnetic resonance molecular imaging of prostate cancer. Mol. Pharm..

[11] Mitsudomi T., Yatabe Y. (2010). Epidermal growth factor receptor in relation to tumor development: EGFR gene and cancer. FEBS J..

[12] Fan Y., Dong Y., Sun X., Wang H., Zhao P., Wang H. (2022). Development and validation of MRI-based radiomics signatures as new markers for preoperative assessment of EGFR mutation and subtypes from bone metastases. BMC Cancer.

[13] Cooper O., Bonert V.S., Rudnick J., Pressman B.D., Lo J., Salvatori R. (2021). EGFR/ErbB2-Targeting lapatinib therapy for aggressive prolactinomas. J. Clin. Endocrinol. Metab..

[14] Wheeler D.L., Dunn E.F., Harari P.M. (2010). Understanding resistance to EGFR inhibitors-impact on future treatment strategies. Nat. Rev. Clin. Oncol..

[15] Chong C.R., Janne P.A. (2013). The quest to overcome resistance to EGFR-targeted therapies in cancer. Nat. Med..

[16] Schneider M.R., Wolf E. (2009). The epidermal growth factor receptor ligands at a glance. J. Cell Physiol.

[17] Cooke R.M., Wilkinson A.J., Baron M., Pastore A., Tappin M.J., Campbell I.D. (1987). The solution structure of human epidermal growth factor. Nature.

[18] Ai S., Duan J., Liu X., Bock S., Tian Y., Huang Z. (2011). Biological evaluation of a novel doxorubicin-peptide conjugate for targeted delivery to EGF receptor-overexpressing tumor cells. Mol. Pharm..

[19] Yang F., Ai W., Jiang F., Liu X., Huang Z., Ai S. (2016). Preclinical evaluation of an epidermal growth factor receptor-targeted doxorubicin-peptide conjugate: toxicity, biodistribution, and efficacy in mice. J. Pharm. Sci..

[20] Rohrer M., Bauer H., Mintorovitch J., Requardt M., Weinmann H.J. (2005). Comparison of magnetic properties of MRI contrast media solutions at different magnetic field strengths. Invest Radiol..

[21] De Leon-Rodriguez L.M., Ortiz A., Weiner A.L., Zhang S., Kovacs Z., Kodadek T. (2002). Magnetic resonance imaging detects a specific peptide-protein binding event. J. Am. Chem. Soc..

[22] Salarian M., Ibhagui O.Y., Yang J.J. (2020). Molecular imaging of extracellular matrix proteins with targeted probes using magnetic resonance imaging. Wiley Interdiscip Rev Nanomed Nanobiotechnol.

[23] Jiang X., Ren M., Shuang X., Yang H., Shi D., Lai Q. (2021). Multiparametric MRI-based radiomics approaches for preoperative prediction of EGFR mutation status in spinal bone metastases in patients with lung adenocarcinoma. J. Magn. Reson. Imaging.

[24] Meng X., Zhao Y., Han B., Zha C., Zhang Y., Li Z. (2020). Dual functionalized brain-targeting nanoinhibitors restrain temozolomide-resistant glioma via attenuating EGFR and MET signaling pathways. Nat. Commun..

[25] Weissleder R. (2006). Molecular imaging in cancer. Science.

[26] Fuchs B.C., Wang H., Yang Y., Wei L., Polasek M., Schuhle D.T. (2013). Molecular MRI of collagen to diagnose and stage liver fibrosis. J. Hepatol..

[27] Townsend T.R., Moyle-Heyrman G., Sukerkar P.A., MacRenaris K.W., Burdette J.E., Meade T.J. (2014). Progesterone-targeted magnetic resonance imaging probes. Bioconjug. Chem..

[28] Zhou Z., Wu X., Kresak A., Griswold M., Lu Z.R. (2013). Peptide targeted tripod macrocyclic Gd(III) chelates for cancer molecular MRI. Biomaterials.

[29] Wilson C.L., Monteith W.B., Danell A.S., Burns C.S. (2006). Purification and characterization of the central segment of prothymosin-alpha: methodology for handling highly acidic peptides. J. Pept. Sci..

[30] Kimura R.H., Miao Z., Cheng Z., Gambhir S.S., Cochran J.R. (2010). A dual-labeled knottin peptide for PET and near-infrared fluorescence imaging of integrin expression in living subjects. Bioconjug. Chem..

[31] Cao L., Li B., Yi P., Zhang H., Dai J., Tan B. (2014). The interplay of T1- and T2-relaxation on T1-weighted MRI of hMSCs induced by Gd-DOTA-peptides. Biomaterials.

[32] Park J.A., Lee Y.J., Ko I.O., Kim T.J., Chang Y., Lim S.M. (2014). Improved tumor-targeting MRI contrast agents: gd(dota) conjugates of a cycloalkane-based RGD peptide. Biochem. Biophys. Res. Commun..

[33] Bai L., Zhu R., Chen Z., Gao L., Zhang X., Wang X. (2006). Potential role of short hairpin RNA targeting epidermal growth factor receptor in growth and sensitivity to drugs of human lung adenocarcinoma cells. Biochem. Pharmacol..

[34] Overholser J.P., Prewett M.C., Hooper A.T., Waksal H.W., Hicklin D.J. (2000). Epidermal growth factor receptor blockade by antibody IMC-C225 inhibits growth of a human pancreatic carcinoma xenograft in nude mice. Cancer.

[35] Jackson A.W., Chandrasekharan P., Shi J., Rannard S.P., Liu Q., Yang C.T. (2015). Synthesis and in vivo magnetic resonance imaging evaluation of biocompatible branched copolymer nanocontrast agents. Int. J. Nanomedicine..

[36] Hyodo F., Eto H., Naganuma T., Koyasu N., Elhelaly A.E., Noda Y. (2022). In Vivo dynamic nuclear polarization magnetic resonance imaging for the evaluation of redox-related diseases and theranostics. Antioxid Redox Signal.

[37] Wu X., Burden-Gulley S.M., Yu G.P., Tan M., Lindner D., Brady-Kalnay S.M. (2012). Synthesis and Evaluation of a Peptide Targeted Small Molecular Gd-DOTA Monoamide Conjugate for MR Molecular Imaging of Prostate Cancer. Bioconjug. Chem..

