# Supplementary material for: EGFR-targeted gadolinium contrast agents for enhanced molecular magnetic resonance imaging of tumors
Source: J Biol Chem. 2025 Nov 29;302(1):110992. doi: 10.1016/j.jbc.2025.110992 (PMC12810539; doi:10.1016/j.jbc.2025.110992)
Supplement: Supplementary Material 1 [file mmc1.docx]

EGFR-Targeted Gadolinium Contrast Agents for Enhanced Molecular Magnetic Resonance Imaging of Tumors

Mengyao Chen^1, 2, ‡,^ Jing Chen^1, 2, ‡^, Xue Ren^1, 2^, Chunping Liu^3, 4, 5^,Chenwu Bai^1, 2^, Xiaoya Wang^1, 2^, Jianli Duan^1, 2^, Shibin Ai^1, 2^, Xinxin Yan^1, 2^, Fan Yang^3, 4, 5,^ *, and Xin Liu^1, 2,^ *

From the ^1^ Department of Colorectal and Anal Surgery, Zhongnan Hospital of Wuhan University, School of Pharmaceutical Sciences, Wuhan University, Wuhan 430071, China; ^2^ Key Laboratory of Combinatorial Biosynthesis and Drug Discovery, Ministry of Education, Wuhan University, Wuhan, China; ^3^Department of Radiology, Union Hospital, Tongji Medical College, Huazhong University of Science and Technology, Wuhan 430022, China; ^4^ Hubei Provincial Clinical Research Center for Precision Radiology & Interventional Medicine, Wuhan 430022, China; ^5^ Hubei Key Laboratory of Molecular Imaging, Wuhan 430022, China.

**
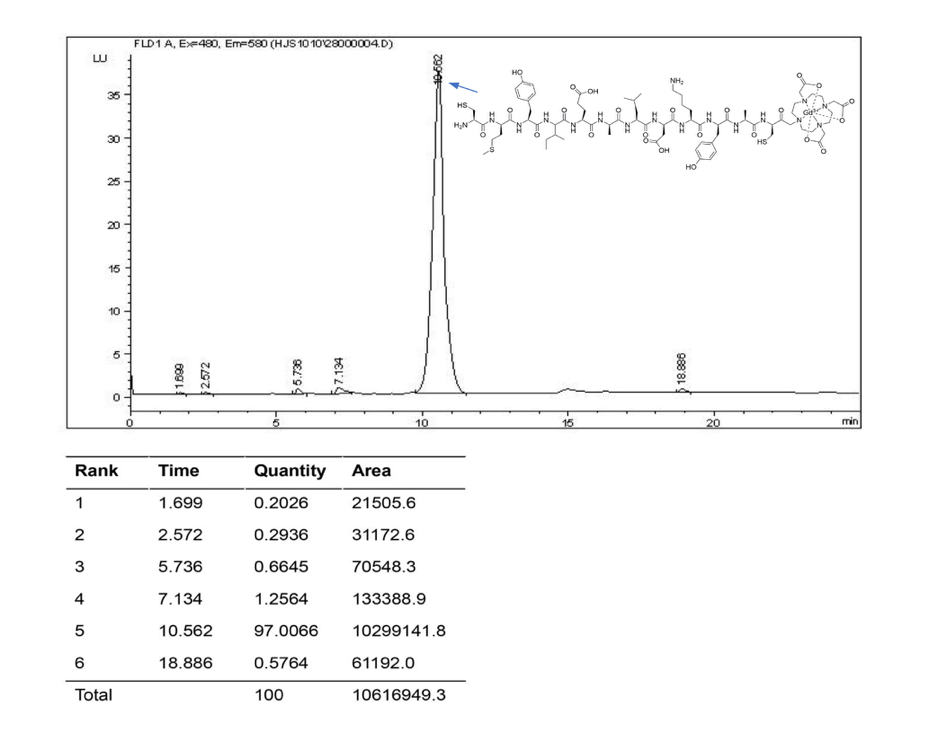
**

**Supplementary** **Figure 1.** HPLC of EBP-Gd-DO3A. The purity of the synthesized EBP-Gd-DO3A was analyzed by high-performance liquid chromatography (HPLC) using an Ultimate 3000 system (Thermo Fisher Scientific, USA).


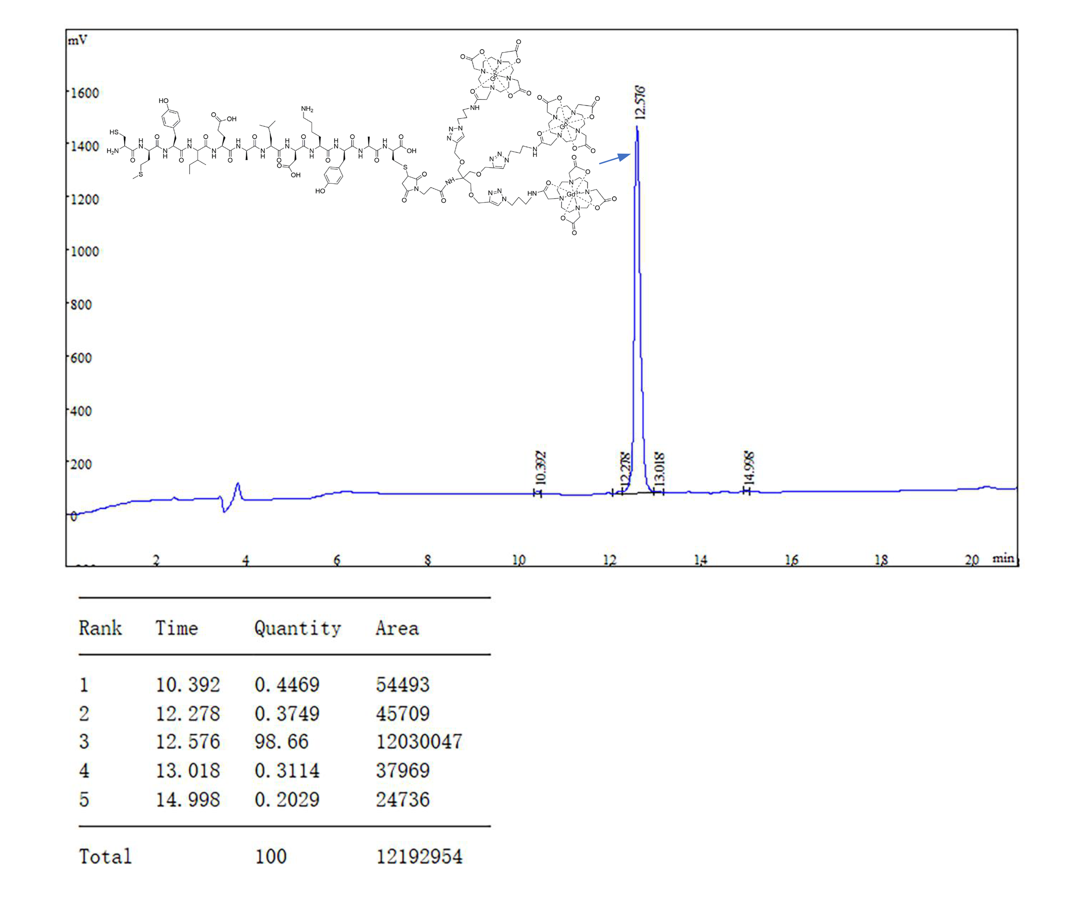
**Supplementary** **Figure 2.** HPLC of EBP-(Gd-DO3A)_3_


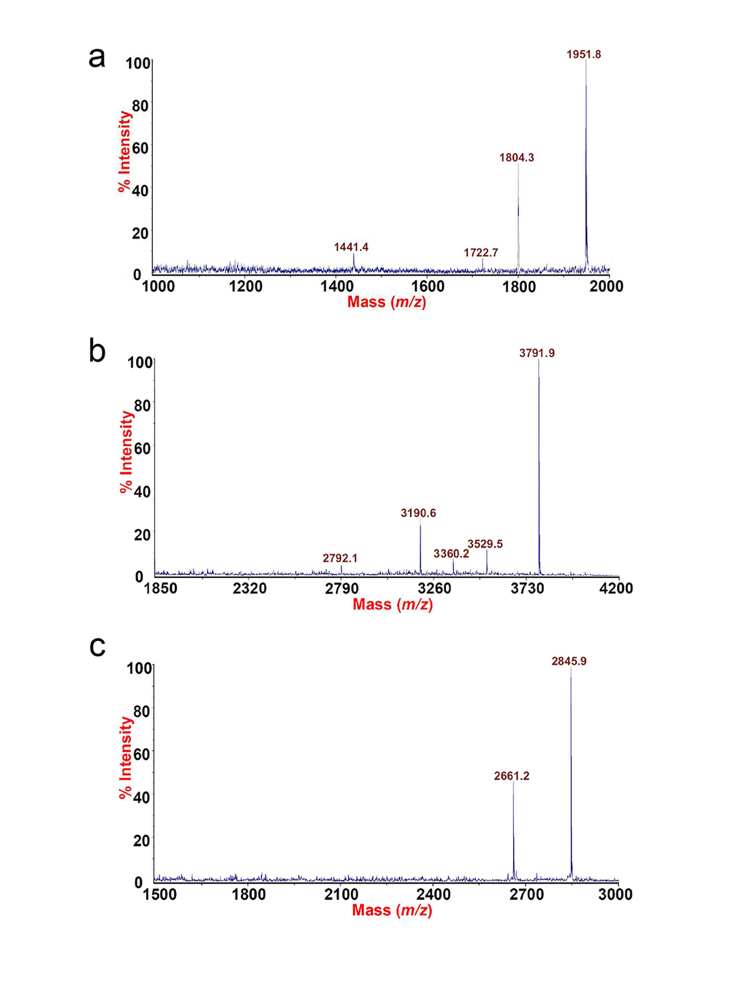


**Supplementary Figure 3.** MALDI-TOF mass spectra of (a) EBP-Gd-DO3A; (b) EBP-(Gd-DO3A)_3_; (c) Cy5.5-EBP-Gd-DO3A. The spectra were recorded on an AB SCIEX 5800 MALDI-TOF mass spectrometer, confirming the expected molecular weights of the synthesized compounds.


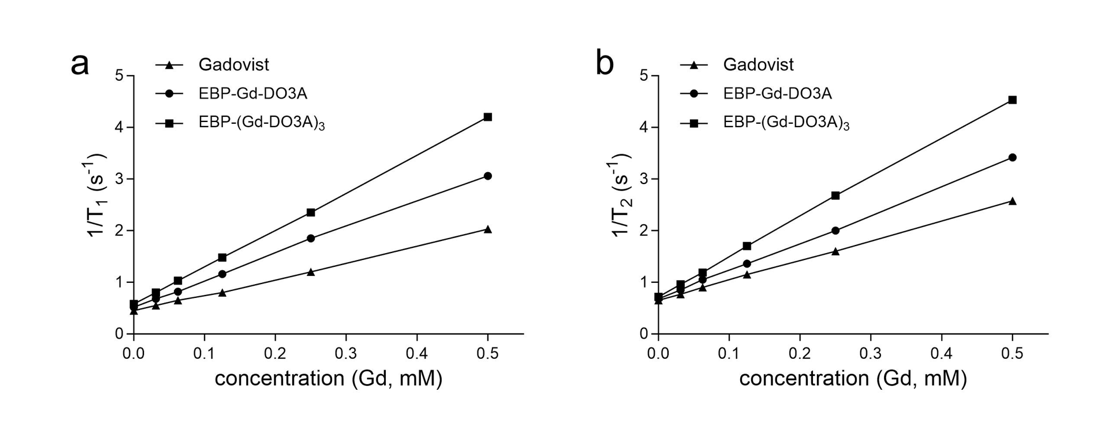
**Supplementary** **Figure 4.** (a) Longitudinal relaxivity (*r_1_*) and (b) transverse relaxivity (*r_2_*) of EBP-Gd-DO3A, EBP-(Gd-DO3A)_3_, and a control (Gadovist^®^) measured in aqueous solutions at 3T.


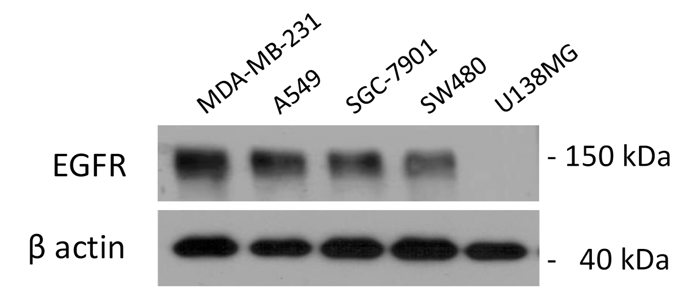


**Supplementary** **Figure 5.** EGFR expression in tumor cells. Cell lysates were prepared from MDA-MB-231 (lane 1), A549 (lane 2), SGC-7901 (lane 3), SW480 (lane 4), and U138MG (lane 5) cells, separated by SDS-PAGE, and analyzed by Western blotting.


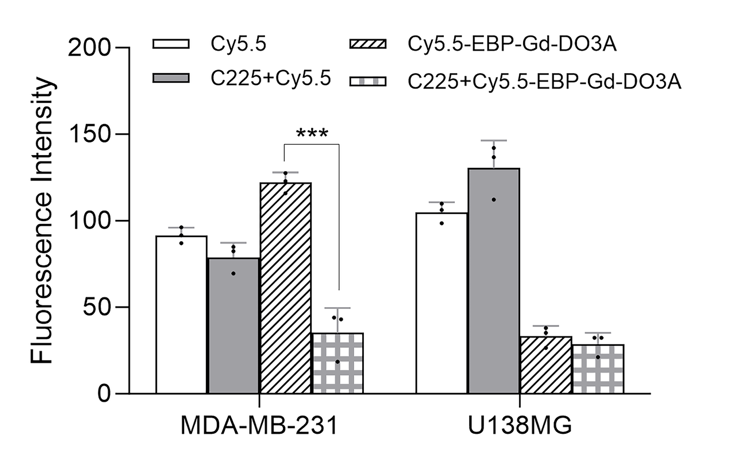


**Supplementary** **Figure 6.** Quantitative analysis of cellular uptake of Cy5.5-labelled agents in MDA-MB-231 and U138MG cells. Fluorescence intensity was quantified from confocal microscopy images. Bars represent the mean ± SD. Statistical significance was determined using two-tailed Student’ s t-test (****p* < 0.001).
