# Supplementary material for: EGFR-targeted gadolinium contrast agents for enhanced molecular magnetic resonance imaging of tumors
Source: J Biol Chem. 2025 Nov 29;302(1):110992. doi: 10.1016/j.jbc.2025.110992 (PMC12810539; doi:10.1016/j.jbc.2025.110992)
Supplement: Supplementary Table 1 Final [file mmc2.docx]

**Supplementary Table 1** Cellular Gd content in selected cell lines after 1 h and 12 h of incubation with EBP-Gd-DO3A, EBP-(Gd-DO3A)_3_ or Gadovist^®^

| Cells | Concentration of cellular Gd (μM/10^6^ cells) | | | | | |
| --- | --- | --- | --- | --- | --- | --- |
|  | 1 h | | | 12 h | | |
|  | Gadovist^®^ | EBP-Gd-DO3A | EBP-(Gd-DO3A)_3_ | Gadovist^®^ | EBP-Gd-DO3A | EBP-(Gd-DO3A)_3_ |
| MDA-MB-231 | 0.18 ± 0.06 | 0.43 ± 0.05 | 1.17 ± 0.12 | 0.13 ± 0.02 | 1.16 ± 0.10 | 2.79 ± 0.18 |
| A549 | 0.25± 0.05 | 0.37 ± 0.07 | 1.04 ± 0.06 | 0.18 ± 0.04 | 1.24 ± 0.19 | 2.66 ± 0.15 |
| SGC-7901 | 0.15 ± 0.05 | 0.35 ± 0.04 | 1.01± 0.08 | 0.16 ± 0.03 | 1.12 ± 0.16 | 2.31 ± 0.14 |
| SW480 | 0.19 ± 0.07 | 0.31 ± 0.06 | 1.05 ± 0.09 | 0.20 ± 0.07 | 1.01 ± 0.13 | 2.65 ± 0.11 |
| U138MG | 0.20 ± 0.04 | 0.09 ± 0.01 | 0.15 ± 0.04 | 0.24 ± 0.05 | 0.25 ± 0.07 | 0.74 ± 0.17 |

Cells were seeded into 100 mm×20 mm cell culture dishes at a density of approximately 1×10^6^ cells per dish and incubated with EBP-Gd-DO3A, EBP-(Gd-DO3A)_3_ or Gadovist^®^ at Gd concentrations of 50 μM at 37°C for the indicated time points. The Gd content in the cell lysates was quantified by ICP-MS. The values are presented as the mean ± SD (n = 6).
